# Prognostic value of a newly identified *MALAT1* alternatively spliced transcript in breast cancer

**DOI:** 10.1038/bjc.2016.123

**Published:** 2016-05-12

**Authors:** Didier Meseure, Sophie Vacher, François Lallemand, Kinan Drak Alsibai, Rana Hatem, Walid Chemlali, Andre Nicolas, Leanne De Koning, Eric Pasmant, Celine Callens, Rosette Lidereau, Antonin Morillon, Ivan Bieche

**Affiliations:** 1 Department of Genetics, Unit of Pharmacogenetics, Institut Curie, 26 rue d'Ulm, Paris Cedex F-75248, France; 2 Department of Pathology, Platform of Investigative Pathology, Institut Curie, 26 rue d'Ulm, Paris Cedex F-75248, France; 3 Department of Translational Research, Institut Curie, 26 rue d'Ulm, Paris Cedex F-75248, France; 4 EA7331, Faculty of Pharmaceutical and Biological Sciences, Paris Descartes University, Sorbonne Paris Cité, Paris Cedex F-75006, France; 5 CNRS UMR 3244, Institut Curie, 26 rue d'Ulm, Paris Cedex F-75248, France

**Keywords:** *MALAT1*, alternatively spliced transcript, breast cancer, prognostic value, signalling pathways

## Abstract

**Background:**

Epigenetic deregulation is considered as a new hallmark of cancer. The long non-coding RNA *MALAT1* has been implicated in several cancers; however, its role in breast cancer is still little known.

**Methods:**

We used RT–PCR, *in situ* hybridisation, and RPPA methods to quantify (i) the full-length (FL) and an alternatively spliced variant (Δsv) of *MALAT1*, and (ii) a panel of transcripts and proteins involved in *MALAT1* pathways, in a large series of breast tumours from patients with known clinical/pathological status and long-term outcome.

**Results:**

*MALAT1* was overexpressed in 14% (63/446) of the breast tumours. *MALAT1*-overexpressed tumour epithelial cells showed marked diffuse nuclear signals and numerous huge nuclear speckles. Screening of the dbEST database led to the identification of Δsv*-MALAT1*, a major alternatively spliced *MALAT1* transcript, with a very different expression pattern compared with FL*-MALAT1*. This alternative Δsv*-MALAT1* transcript was mainly underexpressed (18.8%) in our breast tumour series. Multivariate analysis showed that alternative Δsv*-MALAT1* transcript is an independent prognostic factor. Δsv*-MALAT1* expression was associated with alterations of the pre-mRNAs alternative splicing machinery, and of the Drosha-DGCR8 complex required for non-coding RNA biogenesis. Alternative Δsv*-MALAT1* transcript expression was associated to YAP protein status and with an activation of the PI3K-AKT pathway.

**Conclusions:**

Our results reveal a complex expression pattern of various *MALAT1* transcript variants in breast tumours, and suggest that this pattern of expressions should be taken into account to evaluate *MALAT1* as predictive biomarker and therapeutic target.

Several studies have recently shown that expression of long non-coding RNAs (lncRNAs) are dysregulated in various cancers and that these lncRNAs have important roles in tumourigenesis and tumour progression (Spizzo *et al*, 2012). One example of such oncogenic lncRNA is *HOTAIR*, which is highly expressed in breast cancer and is a predictor for metastasis formation and associated with a poor prognosis (Gupta *et al*, 2010). Among these lncRNAs, *MALAT1* (metastasis-associated lung adenocarcinoma transcript 1), also referred as *NEAT2* (nuclear-enriched abundant transcript 2) was discovered 10 years ago by using a subtractive hybridisation approach. *MALAT1* was originally identified as a transcript showing significant expression in non-small cell lung tumours at high risk for metastasis (Ji *et al*, 2003). *MALAT1* gene has a length of 8708 bp (NR_002819.2) and is localised in chromosome 11q13.1. Unlike most of lncRNAs, *MALAT1* is extremely abundant, ubiquitously expressed and highly conserved among mammals, with potentially major functional roles in mammalian cells. *MALAT1* is a nuclear-retained lncRNA, suggesting both structural and functional properties, for example, nuclear architecture and organisation, splicing, or gene-expression regulation (Gutschner *et al*, 2013a). *MALAT1* has been implicated in alternative splicing regulation, showing interactions with several splicing factors, such as SRSF1 (Tripathi *et al*, 2010). *MALAT1* has also been linked to transcriptional control of genes involved in cell cycle, cell motility and EMT (Gutschner *et al*, 2013a). *MALAT1* could act as a transcription activator by mediating assembly of Polycomb repressive complexes (Yang *et al*, 2011).

*MALAT1* upregulation has been reported in several tumour types and is also a negative prognostic factor in lung, pancreas, colorectal and bladder cancers (Zhang *et al*, 2015). Molecular mechanisms involved in *MALAT1* dysregulation are still unclear. Activation of *MALAT1* by gene amplification seems unlikely because *MALAT1* is located in a chromosomal region (11q13.1) not recurrently amplified in human cancers (Curtis *et al*, 2012). Mutations in the *MALAT1* gene were recently discovery in human cancers (The Cancer Genome Atlas studies; Kandoth *et al*, 2013). *MALAT1* seems the most frequently lncRNA mutated in human cancers. Rare cases of chromosomal translocations involving *MALAT1* have also been reported in mesenchymal harmatomas and renal cell carcinomas (Davis *et al*, 2003; Mathews *et al*, 2013). *MALAT1* epigenetic dysregulation mediated by CpG island methylation was not reported. A post-transcriptional *MALAT1* regulation mechanism mediated by one microRNA (Hsa-miR-125b) has been only reported in bladder cancer (Han *et al*, 2013).

Few studies concerning *MALAT1* in breast cancer are available. Guffanti *et al* (2009) identified *MALAT1* as an abundantly expressed lncRNA in breast tumours. Rare mutations were recently described in luminal breast cancer (Ellis *et al*, 2012). Clinical prognostic value of *MALAT1* dysregulation in breast cancer is little known at this time (Xu *et al*, 2015).

To obtain further insight concerning involvement of *MALAT1* in molecular pathogenesis of breast cancer, we used quantitative real-time reverse-transcriptase–polymerase chain reaction (qRT–PCR) assay, to quantify the full-length (FL) and an alternatively spliced variant (Δsv) of *MALAT1* mRNA expression in a series of 446 patients with unilateral invasive breast tumours and known long-term outcome. We sought links between *MALAT1* mRNA expression pattern and classical clinical and pathological parameters, including patient outcome. We also sought relationships between *MALAT1* and genes and proteins expression known to be involved in different steps of *MALAT1* pathway dysregulation observed in others types of human cancers.

## Materials and methods

### Patients and samples

Samples of 446 unilateral invasive primary breast tumours excised from women managed at Institut Curie–Hôpital René Huguenin (Saint-Cloud, France) from 1978 to 2008 have been analysed. All patients cared in our institution before 2007 were informed that their tumour samples might be used for scientific purposes and had the opportunity to decline. Since 2007, patients treated in our institution have given their approval by signed inform consent. This study was approved by the local ethics committee (Breast Group of René Huguenin Hospital). Samples were immediately stored in liquid nitrogen until RNA extraction. A tumour sample was considered suitable for our study if the proportion of tumour cells exceeded 70%.

All patients (mean age 61.8 years, range 31–91 years) met the following criteria: primary unilateral nonmetastatic breast carcinoma for which complete clinical, histological and biological data were available; no radiotherapy or chemotherapy before surgery; and full follow-up at Institut Curie–Hospital René Huguenin.

Treatment (information available for 438 patients) consisted of modified radical mastectomy in 278 cases (63.9%) and breast-conserving surgery plus locoregional radiotherapy in 160 cases (36.1%). The patients had a physical examination and routine chest radiotherapy every 3 months for 2 years, then annually. Mammograms were done annually. Adjuvant therapy was administered to 360 patients, consisting of chemotherapy alone in 87 cases, hormone therapy alone in 172 cases and both treatments in 101 cases. The histological type and the number of positive axillary nodes were established at the time of surgery. The malignancy of infiltrating carcinomas was scored according to Scarff Bloom Richardson's histoprognostic system.

Hormone receptor (HR; i.e., oestrogen receptor-alpha (ERα), progesterone receptor (PR) and human epidermal growth factor receptor 2 (ERBB2) statuses were determined at the protein level by using biochemical methods (dextran-coated charcoal method, enzyme immunoassay or immunohistochemistry) and confirmed by qRT–PCR assays (Bieche *et al*, 1999, 2001).

The population was divided into four groups according to HRs (ERα and PR) and ERBB2 status, as follows: two luminal subtypes (HR^+^/ERBB2^+^ (*n*=45)) and (HR^+^/ERBB2^−^ (*n*=195)); an ERBB2^+^ subtype (HR^−^/ERBB2^+^ (*n*=46)) and a triple-negative subtype (HR^−^/ERBB2^−^ (*n*=64)). Standard prognostic factors of this tumour set are presented in (Supplementary Table 1). During a median follow-up of 9.1 years (range 4.3 months to 33.2 years), 176 patients developed metastasis.

Ten specimens of adjacent normal breast tissue from breast cancer patients and normal breast tissue from women undergoing cosmetic breast surgery were used as sources of normal RNA.

### RNA extraction

Total RNA was extracted from breast tissue samples by using acid–phenol guanidium. RNA quality was determined by electrophoresis through agarose gels, staining with ethidium bromide and visualisation of the 18S- and 28S-RNA bands under ultraviolet light.

### qRT–PCR

Quantitative values were obtained from the cycle number (Ct value) at which the increase in the fluorescence signal associated with exponential growth of PCR products started to be detected by the laser detector of the ABI Prism 7900 sequence detection system (Perkin-Elmer Applied Biosystems, Foster City, CA, USA), using PE biosystems analysis software according to the manufacturer's manuals.

The precise amount of total RNA added to each reaction mix (based on optical density) and its quality (i.e., lack of extensive degradation) are both difficult to assess. Therefore, transcripts of the *TBP* gene (Genbank accession NM_003194) encoding the TATA box-binding protein (a component of the DNA-binding protein complex TFIID) were also quantified as an endogenous RNA control. Each sample was normalised on the basis of its *TBP* content. *TBP* was selected as an endogenous control due to the absence of known *TBP* retropseudogenes (retropseudogenes lead to co-amplification of contaminating genomic DNA and thus interfere with qRT–PCR, despite the use of primers in separate exons; Bieche *et al*, 1999).

Results, expressed as N-fold differences in target-gene expression relative to the *TBP* gene and termed ‘N*target*', were determined as N*target*=2^ΔCtsample^, where the ΔCt value of the sample was determined by subtracting the average Ct value of target gene from the average Ct value of *TBP* gene.

The target-gene values of the breast tumour samples were subsequently normalised such that the median of the target-gene values for the 10 normal breast tissues was 1.

The primers for *TBP, MALAT1* and others target genes were chosen with the assistance of the Oligo 6.0 program (National Biosciences, Plymouth, MN, USA; Supplementary Table 2). dbEST and nr databases were scanned to confirm the total gene specificity of the nucleotide sequences chosen for the primers and the absence of single-nucleotide polymorphisms. To avoid amplification of contaminating genomic DNA, one of the two primers was placed at the junction between two exons or on two different exons. Agarose gel electrophoresis was used to verify the specificity of PCR amplicons. The conditions of cDNA synthesis and PCR were as previously described (Bieche *et al*, 1999).

### *In situ* hybridisation

We used Stellaris FISH Probes, Human MALAT1 with Quasar 570 Dye (Biosearch Technologies, Petaluma, CA, USA). First, paraffin-embedded tissue sliced at 4–5 *μ*m thickness were obtained from normal and tumour tissues by using a microtome (Thermo scientific Sandom HE 340 E, Walldorf, Germany). Formalin-fixed paraffin-embedded breast tissue sections were deparaffinized by using 100% xylene, 100% ethanol, 95% ethanol, 70% ethanol and RNase-free PBS. Then, slides were incubated for 20 min at 37 °C, and washed twice with PBS. We created a working probe solution at 125 nM (probe diluted in hybridisation buffer). We immersed tissue sections in a wash buffer for 2–5 min, while assembling the humidified chamber. Then, we dispensed 100 *μ*l of working probe solution onto tissue sections, placed them in the humidified chamber, and covered them with parafilm. We incubated tissue sections in the dark at 37 °C for at least 4 h. After decanting wash buffer, we added DAPI nuclear stain and immersed tissue sections in SSC. Finally, we added a small drop of antifade onto tissue sections and covered with a cover glass and proceeded to imaging.

### RPPA

Samples were disrupted in Laemmli buffer (50 mM Tris pH=6.8, 2% SDS, 5% glycerol, 2 mM DTT, 2.5 mM EDTA, 2.5 mM EGTA, 1 × HALT phosphatase inhibitor (Perbio, Villebon-sur-Yvette, France; 78420), protease inhibitor cocktail complete MINI EDTA-free (Roche, Basel, Switzerland; 1836170, 1 tablet per 10 ml), 4 mM Na3VO4 and 20 mM NaF) qsp 5 ml H2O, using a Tissue Lyser (Qiagen, Venlo, Netherlands) and two 5 mm stainless beads per sample. Extracts were then boiled for 10 min at 100 °C, passed through a fine needle to reduce viscosity and centrifuged for 15 min at 13 000 r.p.m. The supernatant was collected and stored at −80 °C. Protein concentration was determined (Pierce BCA reducing agent compatible kit, Pierce, Waltham, MA, USA; ref 23252). Samples were deposited onto nitrocellulose covered slides (Fast slides, Maine Manufacturing, Sanford, ME, USA) using a dedicated arrayer (2470 arrayer, Aushon Biosystems, Billerica, MA, USA). Five serial dilutions, ranging from 1000 to 62.5 *μ*g ml^−1^, and two technical replicates per dilution were printed for each sample. Arrays were labelled with specific antibodies or without primary antibody (negative control), using an Autostainer Plus (Dako, Glostrup, Denmark). Briefly, slides were incubated with avidin, biotin and peroxides blocking reagents (Dako) before saturation with TBS containing 0.1% Tween-20 and 5% BSA (TBST-BSA). Slides were then probed overnight at 4 °C with primary antibodies diluted in TBST-BSA. After washes with TBST, arrays were probed with horseradish peroxidase-coupled secondary antibodies (Jackson ImmunoResearch Laboratories, New market, UK) diluted in TBST-BSA for 1 h at room temperature (RT). To amplify the signal, slides were incubated with Bio-Rad Amplification Reagent (Bio-Rad, Hercules, CA, USA) for 15 min at RT. The arrays were washed with TBST, probed with Alexa647-Streptavidin (Molecular Probes, Eugene, OR, USA) diluted in TBST-BSA for 1 h at RT and washed again in TBST. For staining of total protein, arrays were incubated 15 min in 7% acetic acid and 10% methanol, rinsed twice in water, incubated 10 min in Sypro Ruby (Invitrogen, Carlsbad, CA, USA) and rinsed again. The processed slides were dried by centrifugation and scanned using a GenePix 4000B microarray scanner (Molecular Devices, Sunnyvale, CA, USA). Spot intensity was determined with MicroVigene software (VigeneTech Inc., Carlisle, MA, USA). All primary antibodies used in RPPA have been previously tested by Western Blotting to assess their specificity for the protein of interest.

Raw data were normalised using Normacurve (Troncale *et al*, 2012), which normalises for fluorescent background per spot, a total protein stain and potential spatial bias on the slide. Next, each RPPA slide was median centred and scaled (divided by median absolute deviation). We then corrected for remaining sample loadings effects individually for each array by correcting the dependency of the data for individual arrays on the median value of each sample over all arrays using a linear regression.

### Statistical analysis

The distributions of target mRNA levels were characterised by their median values and ranges. Relationships between mRNA levels of the different target genes, and between mRNA levels and clinical parameters, were identified using nonparametric tests, namely the *χ*^2^-test (relation between two qualitative parameters), the Mann–Whitney's *U*-test (relation between one qualitative parameter and one quantitative parameter) and the Spearman's rank correlation test (relation between two quantitative parameters). Differences were considered significant at confidence levels >95% (*P*<0.05).

To visualise the efficacy of a molecular marker (*MALAT1* level) to discriminate two populations (patients that developed/did not develop metastases) in the absence of an arbitrary cut-off value, data were summarised in an receiver operating characteristic curve (Hanley and McNeil, 1982). The AUC (area under curve) was calculated as a single measure for discriminate efficacy. Metastasis-free survival (MFS) was determined as the interval between initial diagnosis and detection of the first metastasis. Survival distributions were estimated by the Kaplan–Meier method, and the significance of differences between survival rates were ascertained with the log-rank test. The Cox-proportional hazards regression model was used to assess prognostic significance and the results are presented as hazard ratios and 95% confidence intervals.

## Results

### *MALAT1* expression in breast tumours and relationship with classical clinico-pathological parameters and patient outcome

In order to determine the prognostic significance of *MALAT1* expression pattern in human breast tumours, we analysed *MALAT1* mRNA levels in a large series of 446 primary breast tumours from patients with known clinical/pathological status and long-term outcome (Supplementary Table 1).

Among the 446 breast tumour RNA samples tested, 63 (14.1%) tumours showed *MALAT1* mRNA overexpression (N*MALAT1* from 3.02 to 13.4), and only 13 (2.9%) tumours showed *MALAT1* mRNA underexpression (N*MALAT1* from 0.15 to 0.32), as compared with normal breast tissues. We sought links between *MALAT1* mRNA level status and standard clinico-pathological and biological factors in breast cancer (Supplementary Table 3). Significant positive associations were observed between the tumour group showing *MALAT1* overexpression and ERα-positive (*P*=0.000015), PR-positive (*P*=0.00079) and molecular subtypes (*P*=0.00000075). It is noteworthy that majority (9/13) of the *MALAT1* underexpressed tumours was of triple-negative (HR^−^/ERBB2^−^) subtype.

We also examined *PIK3CA* mutation status, and expressions of *EGFR* and *MKI67* (which encodes the proliferation-related antigen Ki-67). None of these three markers showed significant link with *MALAT1* expression.

To further investigate whether *MALAT1* mRNA expression could be of prognostic relevance, the log-rank test was used to identify relations between MFS and *MALAT1* mRNA expression. Results showed that MFS was not significantly influenced by *MALAT1* overexpression status (*P*=0.23; data not shown).

**Figure 1:**
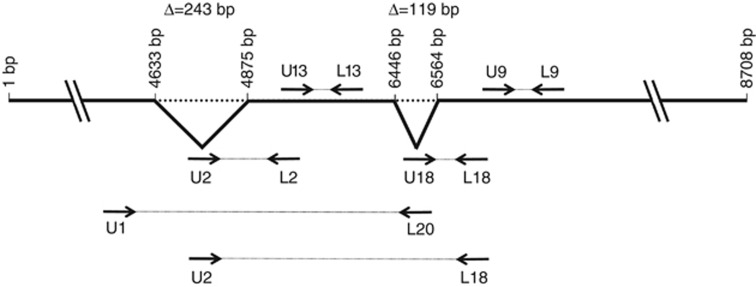
**Location of primers used for *MALAT1* mRNA expression analysis.**Alternative splicing variants were amplified using primer pairs with one of the two primers placed at the junction of the two spliced regions: U2/L2 for the 243 bp alternatively spliced *MALAT1* transcript (*Δ2sv-MALAT1*) and U18/L18 for the 119 bp alternatively spliced *MALAT1* transcript *(Δ1sv-MALAT1*).

Results of *MALAT1* mRNA levels shown in Supplementary Table 3 were obtained by using a primer pair (U13/L13) that encompass region 4891–4975 of published *MALAT1* cDNA sequence (GenBank #NR_002819.2; Figure 1). Similar results (frequency of *MALAT1* overexpression links with classical clinico-pathological parameters and patient outcome) were obtained with a second primer pair (U9/L9) localised in *MALAT1* gene at region 6565–6658 (Figure 1).

### Relationship between *MALAT1* mRNA level and *HOTAIR* and *ANRIL* mRNA levels

We tested possible relation between *MALAT1* and *HOTAIR* and *ANRIL* (the two most documented lncRNAs that also interact with Polycomb repressive complexes) mRNA levels. We did not observe any association between *MALAT1* and these two Polycomb complexes associated lncRNAs (*r*=+0.069, *P*=0.14 for *HOTAIR*; *r*=+0.014, *P*=0.76 for *ANRIL*; Spearman's rank correlation test).

### Localisation of *MALAT1* transcript in epithelial tumour cells

**Figure 2:**
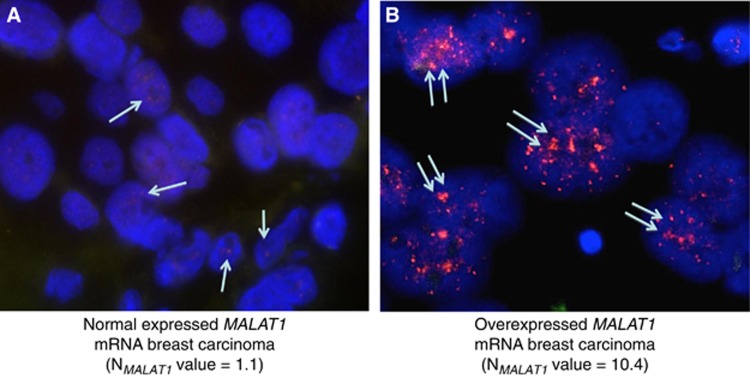
**ISH of *MALAT1* RNA in breast tumours.**(**A**) Example of normal-expressed *MALAT1* mRNA breast tumour (N_*MALAT1*_ value=1.1, as determined by qRT–PCR analysis). Weak signals represented by small speckles (speckles in red, arrow) of equivalent size and shape, regularly distributed within nuclei of tumour cells (nuclei in blue) × 400. (**B**) Example of overexpressed *MALAT1* mRNA breast tumour (N_*MALAT1*_ value=10.4). Marked diffuse signals and numerous and frequently huge nuclear speckles (speckles in red, two arrows) of variable size and shape within nuclei of tumour cells (nuclei in blue) × 600.

We detected specific *MALAT1* RNA in epithelial and stromal cells of all ten tumour samples studied by *in situ* hybridisation (ISH). *MALAT1* mRNA was found exclusively in the nucleus of both stromal and tumour epithelial cells. We detected strong specific *MALAT1* RNA level in epithelial cells of the five tumours, which overexpressed *MALAT1* mRNA (using qRT–PCR analysis) and low specific *MALAT1* RNA level in the five tumours which did not overexpress *MALAT1* mRNA. We thus obtained a perfect match between *MALAT1* mRNA expression by using qRT–PCR and ISH analysis. *MALAT1*-overexpressed tumour epithelial cells showed marked diffuse nuclear signals and numerous nuclear speckles of variable size and shape as compared with *MALAT1* normal-expressed tumour epithelial cells (Figure 2).

### Identification of a major alternatively spliced *MALAT1* transcript in breast tumours, and relationships with classical clinico-pathological parameters and patient outcome

Screening of the dbEST database with the *MALAT1* cDNA led to identification of two major groups of alternatively spliced *MALAT1* ESTs. The first major alternatively spliced *MALAT1* transcript (named *Δ1sv*-*MALAT1*) had a 119-bp deletion (from 6446 to 6564, from the NR_002819.2 sequence), resulting from alternative splicing of *MALAT1* mRNA, whereas the second alternatively spliced *MALAT1* transcript (named *Δ2sv*-*MALAT1*) had a 243-bp deletion (from 4633 to 4875, from the NR_002819.2 sequence). The deleted nucleotide sequences show consensus sequences of donor/acceptor splice sites.

To verify presence and quantify mRNA levels of these alternative splicing variants in our breast cancer series, we carried out non-quantitative (classical) RT–PCR using primer pairs with one of the two primers placed at the junction of the two spliced regions: U2/L2 for the 243-bp alternatively spliced *MALAT1* transcript *(Δ2sv*-*MALAT1*) and U18/L18 for the 119-bp alternatively spliced *MALAT1* transcript *(Δ1sv*-*MALAT1*; Figure 1). However, additional qualitative analyses using primer couples U1/L20 or U2/L18 (Figure 1 and Supplementary Figure 1) showed that these two splices were always associated together. This unique transcript, showing both 119-bp and 243-bp deletions, is named Δsv*-MALAT1* for the remaining part of the manuscript, and FL*-MALAT1* for the FL transcript.

**Figure 3:**
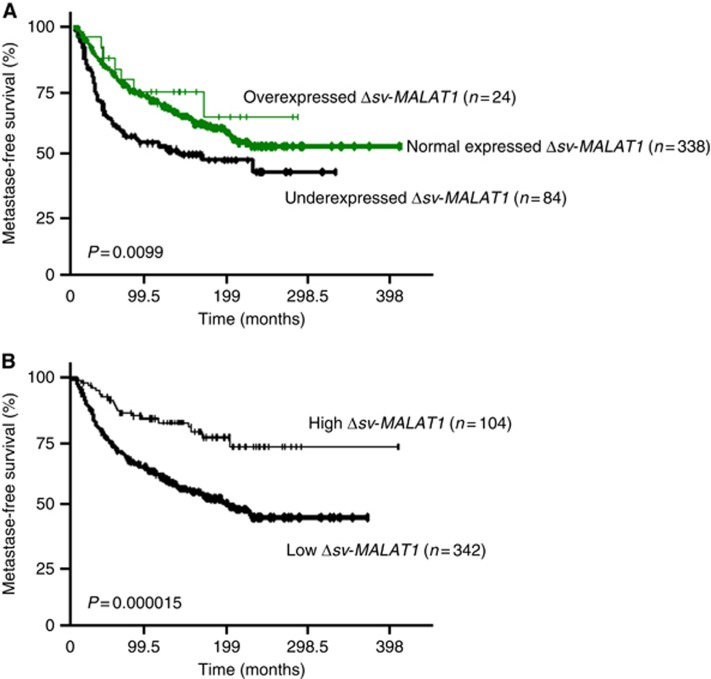
**MFS curves of patient groups according to Δsv*-MALAT1* mRNA expression level in the series of 446 breast tumours.**(**A**) MFS curves of three patients groups with under, normal and overexpressed Δsv*-MALAT1* tumours, as compared with normal breast tissues. (**B**) MFS curves for patients with high-Δsv-*MALAT1*-expressing and low-*MALAT1*-expressing tumours, using an optimal cut-off value.

All the 446 breast tumour RNA samples tested showed a marked presence of Δsv*-MALAT1* transcript. We observed a highly positive correlation between Δsv*-MALAT1* and FL*-MALAT1* expressions (Supplementary Figure 2A). The relative expression of Δsv*-MALAT1* and FL*-MALAT1* for each individual sample listed as dot/box plots is indicated in Supplementary Figure 2B. As compared with normal breast tissues, 24 (5.4%) tumours showed Δsv*-MALAT1* mRNA overexpression (NΔsv*-MALAT1* from 3.16 to 8.4), and surprisingly 84 (18.8%) tumours showed Δsv*-MALAT1* mRNA underexpression (NΔsv*-MALAT1* from 0.05 to 0.32). Marked significant positive associations were observed between the tumour group showing Δsv*-MALAT1* underexpression and large macroscopic tumour size (*P*=0.0023), ERα-negative (*P*=0.000062), PR-negative (*P*=0.0000051) and molecular subtypes (*P*=0.00074; Table 1). We observed the same associations between Δsv*-MALAT1* mRNA levels and molecular subtypes in a series of 21 breast cell lines, including 4 non-cancerous cell lines, 8 triple-negative cell lines, 4 ERBB2 cell lines and 5 RE^+^ cell lines (Supplementary Table 4). In particular, we observed a marked Δsv-MALAT1 underexpression in the triple-negative subtype. Δsv-*MALAT1* underexpression was also highly associated with *MKI67* mRNA levels (*P*=0.00033). MFS was significantly influenced by Δsv-*MALAT1* (overexpression *vs* normal expression *vs* underexpression) expression status (*P*=0.0099; Figure 3A). AUC analyses was then performed to identify a putative cut-point to divide the cohort into two relevant Δsv-*MALAT1* expression subgroups. Results confirmed that MFS of patients with low Δsv-*MALAT1*-expressing tumours (5-year RFS 70.3±2.5% 10-year RFS 58.5±2.8% 15-year RFS 51.4±3.0%) was shorter than that of patients whose tumours highly expressed Δsv-*MALAT1* (5-year RFS 85.9±3.5% 10-year RFS 82.3±3.9% 15-year RFS 76.0±5.1% *P*=0.000015; Figure 3B).

Results of Δsv-*MALAT1* mRNA levels shown in Table 1 were obtained by using a primer pair (U18/L18) that encompasses the 119 bp deleted region. Similar results (frequency of Δsv*-MALAT1* overexpression, links with classical clinico-pathological parameters and patient outcome) were obtained with a second primer pair (U2/L2) that encompass the 243 bp deleted region (Figure 1; Supplementary Table 5).

Finally, the prognostic significance of the five parameters identified in univariate analysis, including histopathological grade, lymph node status, macroscopic tumour size, PR status (Supplementary Table 1) and Δsv-*MALAT1* expression status (Figure 3B) persisted (except for lymph node and PR status) in Cox multivariate regression analysis of MFS (Supplementary Table 6).

### Relationship between Δsv-*MALAT1* mRNA levels and *Hsa-miR-125b* expression status

As *Hsa-miR-125b* suppresses bladder cancer development by downregulating *MALAT1* (Han *et al*, 2013), we tested the possible negative correlation between Δsv-*MALAT1* and *Hsa-miR-125b* mRNA level in breast cancer. *Hsa-miR-125b* levels were analysed in 20 low-Δsv-*MALAT1*-expressing (median mRNA value: 0.36) and 20 high-Δsv-*MALAT1-*expressing breast tumours (median mRNA value: 2.13). We found no link between Δsv-*MALAT1* and *Hsa-miR-125b* expression status: the median *Hsa-miR-125b* value was 0.13 in low-Δsv-*MALAT1*-expressing breast tumours and 0.11 in high-Δsv-*MALAT1*-expressing breast tumours. Similar results were obtained with 20 low-FL*-MALAT1*-expressing and 20 high-FL*-MALAT1*-expressing breast tumours.

### Relationship between Δsv-*MALAT1* mRNA levels and YAP protein level

As YAP protein regulates transcription of *MALAT1* gene in liver cancer (Wang *et al*, 2014), we tested the possible positive correlation between YAP protein and Δsv-*MALAT1* mRNA levels in breast cancer. YAP protein levels were analysed by using RPPA assay in 143 samples from our series of 446 breast tumours. We found a significant positive link with Δsv*-MALAT1* mRNA level (*r*=+0.303, *P*=0.00032; Spearman's rank correlation test) but no link between YAP protein and FL*-MALAT1* mRNA levels of expression. As Δsv*-MALAT1* low level is associated with a poor outcome and with low-YAP-protein level, we tested if YAP protein level could be also of prognostic relevance. We did not observe in our smaller series of 143 samples, any statistical correlation between low-YAP-protein expression and poor outcome.

### Relationship between Δsv-*MALAT1* mRNA levels and a large panel of selected genes involving in various signalling pathways

To obtain further insight into *MALAT1* dysregulated pathways in breast cancer, we evaluated by qRT–PCR mRNA expression of a large number of selected genes in 20 low-Δsv-*MALAT1*-expressing and 20 high-Δsv-*MALAT1*-expressing breast tumours. We assessed expression level of 48 genes involved in various cellular and molecular phenomena associated with carcinogenesis. These genes encode proteins involved in cell cycle control (*n*=7), cell migration (*n*=5), polycomb repressive complexes (PRC1) (*n*=3) and PRC2 (*n*=5), EMT (*n*=7), apoptosis (*n*=6) and DNA repair (Yang *et al*, 2011). We also focused on expression of well-known regulators (DGCR8; AGO2) and interactors (SRSF1, SRSF2, SRSF3, UHMK1) of *MALAT1* (Gutschner *et al*, 2013a), as well as transcriptional dysregulated genes after *MALAT1* depletion in A549 lung adenoma cell line (ROBO1, MCAM; Gutschner *et al*, 2013b) and HeLa cells (IFI44; Miyagawa *et al*, 2012).

Expression of 19 (39.5%) of these 48 genes was significantly positively associated with Δsv-*MALAT1* expression (Table 2). Genes significantly associated to Δsv-*MALAT1* were mainly involved in cell migration (*RHOB, PLAU/UPA* and *MMP14*), Polycomb repressive complex PRC2 (*EED, SUZ12, JARID2, TUG1*), apoptosis (*BIRC6*), DNA repair (*ATM, MSH2, XRCC1*) and regulators (*DGCR8*) and interactors (*SRSF1, SRSF3, UHMK1*) of *MALAT1*. Genes involved in cell cycle control and EMT, as well as putative *MALAT1*-inducible genes (identified by *MALAT1* depletion in cell lines) were not linked to the *MALAT1* in breast cancer.

### Relationship between levels of Δsv-*MALAT1* mRNA and RTK/MAPK/PI3K proteins

As several studies recently suggested that *MALAT1* promotes proliferation and metastasis of various cancers by activating the RTK/MAPK/PI3K pathways (Wu *et al*, 2014; Dong *et al*, 2015; Xu *et al*, 2015), we tested possible correlation between Δsv-*MALAT1* and various proteins involved in these signalling pathways.

Twenty-eight protein (non-phosporylated or/and phosphorylated) levels were analysed using RPPA assays in 143 samples from our series of 446 breast tumours. These selected proteins are involved in TKR (*n*=9), MAPK (*n*=4) and PI3K/AKT (*n*=15) pathways (Table 3). Low-Δsv-*MALAT1* mRNA level were associated to high levels of 4 among the 15 proteins involved in the PI3K/AKT pathway (i.e., FOXO1, p70 S6 Kinase total protein and phosphorylated in Threonine 389, S6 ribosomal protein phosphorylated in Ser240/Ser244), but to none of the two others signalling pathways. Low-FL*-MALAT1* mRNA level was exclusively associated to high level of FOXO1 (Table 3).

## Discussion

Recent studies have demonstrated the importance of non-protein-coding part of human genome in carcinogenesis. Among numerous kinds of non-protein-coding RNAs, lncRNAs have a key regulatory role in cancer biology. LncRNAs are dysregulated in different types of cancer and the expression levels of certain lncRNAs are associated with metastasis and prognosis of cancer. Overexpression of certain lncRNAs, behaving like oncogenes, can promote tumour growth and cancer cell invasion (Cheetham *et al*, 2013).

In this study, we focused on the lncRNA *MALAT1* that has been shown dysregulated in various cancer types (Zhang *et al*, 2015), but poorly studied in breast cancer. One study, using deep-sequencing technology, identified *MALAT1* as one of the highly expressed lncRNAs in breast tumours (Guffanti *et al*, 2009).

We tested 10 normal breast tissue RNAs and 446 unilateral invasive primary breast tumour RNAs, using qRT–PCR method. *MALAT1* mRNA was detected in all breast tumour samples and also in all normal breast tissues.

Overexpression of *MALAT1* mRNA was detected in 14% (63/446) of breast tumours, confirming the oncogenic role of *MALAT1*. Indeed, *MALAT1* is overexpressed in several cancer types, including lung, colon and hepatocarcinoma, and overexpression of *MALAT1* in various cell lines enhanced cell proliferation, whereas in nude mice, increased levels of *MALAT1* promoted tumour formation (Ji *et al*, 2003; Guo *et al*, 2010; Gupta *et al*, 2010; Gibb *et al*, 2011; Schmidt *et al*, 2011; Lai *et al*, 2012). Additional studies have also demonstrated that depletion of *MALAT1* impaired proliferative and invasiveproperties of cancer cells (Guo *et al*, 2010, Schmidt *et al*, 2011, Gutschner *et al*, 2013b).

By using ISH, we showed that *MALAT1* transcripts were predominantly localised in nuclear speckles. Nucleus of the *MALAT1*-overexpressed tumour epithelial cells showed marked diffuse nuclear signals and numerous huge nuclear speckles.

No significant links were observed between *MALAT1* mRNA overexpression and markers of aggressiveness, including histopathological grade, lymph node status and macroscopic tumour size, suggesting that overexpression of *MALAT1* does not have a major role in aggressiveness of breast carcinomas. Moreover, we observed a link between *MALAT1* mRNA overexpression and HR-positive tumours (a marker of good prognostic), suggesting that *MALAT1* could be an ER-induced gene in breast cancer. Finally, survival analysis did not reveal that patients with *MALAT1-*overexpressed tumour had shorter MFS.

Alternative mRNA splicing is a common mechanism for regulating gene expression in higher eukaryotes, and there are many examples of development-specific, tissue-specific and tumour-specific differences in splicing events. In the GENCODE v7 catalogue of human lncRNAs, >25% of lncRNA genes show evidence of alternative splicing with at least two different transcript isoforms per gene locus (Derrien *et al*, 2012). The vast majority of alternatively spliced lncRNA introns are flanked by canonical splice sites (GT/AG), with no differences in splicing signal compared with the protein-coding genes (Derrien *et al*, 2012). In the present study, by screening the dbEST database with the FL-*MALAT1* cDNA (named FL*-MALAT1*), we identified a major alternatively spliced *MALAT1* transcript (named Δsv-*MALAT1*) with two concomitant deleted regions of 119 bp and 243 bp. As expected, these alternatively spliced sequences showed consensus sequences of donor/acceptor splice sites. In our cohort, Δsv-*MALAT1* showed a very different expression pattern, as compared with FL*-MALAT1*. Indeed, Δsv-*MALAT1* expression varied widely in tumour tissues, being both underexpressed (18.8%) and overexpressed (5.4%). Surprisingly, a significant link was observed between Δsv-*MALAT1* underexpression and tumours with large macroscopic size, negative for HRs and expressing high *MKI67* mRNA levels, suggesting that underexpression of Δsv-*MALAT1* has a role in aggressiveness of breast tumours. In this regard, in contrast to the FL-*MALAT1* expression, survival analysis revealed that patients with low-Δsv-*MALAT1*-expressed tumours had shorter MFS. Moreover, multivariate analysis showed that Δsv-*MALAT1* expression status was an independent prognostic marker for MFS. This alternatively spliced *MALAT1* transcript isoform could act as decoys, sequestering biomolecules that fixed on the FL-*MALAT1* transcript and thus dysregulating its function. Taken together, these results suggest that this alternatively spliced Δsv-*MALAT1* transcript isoform has a significant contribution to overall *MALAT1* function and breast carcinogenesis.

Further studies are necessary to elucidate the genetic (or epigenetic) mechanisms responsible for the observed underexpression of Δsv-*MALAT1*, in breast cancer. It is unlikely that gene amplification is one of the mechanisms for *MALAT1* overexpression because *MALAT1* is located in a chromosomal region (11q13.1) non-recurrently amplified in breast cancer (Curtis *et al*, 2012; Zack *et al*, 2013). Mutations in the *MALAT1* gene, recently discovered in human cancers, are rare in breast (1.1%) as compared with other cancer types such as bladder cancer (15.3%) (Kandoth *et al*, 2013). *MALAT1* epigenetic dysregulation mediated by CpG island methylation is not reported till date. In the present study, post-transcriptional regulation of *MALAT1* by Hsa-miR-125b as described in bladder cancer (Han *et al*, 2013) was not observed in our breast tumour series. Conversely, our data suggested a transcriptional regulation of the Δsv-*MALAT1* (but not of the FL-*MALAT1*) by the transcriptional co-activator YAP, as described in liver cancer (Wang *et al*, 2014). Further studies are necessary, using functional assays, to confirm this association. In the downstream *MALAT1* pathway, our results suggested that Δsv-*MALAT1* (but not FL*-MALAT1*) could activate the PI3K/AKT pathway, in partial agreement with previous data (Wu *et al*, 2014; Dong *et al*, 2015; Xu *et al*, 2015).

We also assessed the expression levels of gene panel putatively involved in various cellular and molecular phenomena associated with carcinogenesis via dysregulation of Δsv-*MALAT1*. These genes encode proteins involved in cell cycle control, cell migration, polycomb repressive complexes (PRC1 and 2), EMT, apoptosis and DNA repair, as well as regulators and interactors of Δsv-*MALAT1*, or known *MALAT1*-induced genes (Miyagawa *et al*, 2012; Gutschner *et al*, 2013b). We identified a strong positive link between Δsv-*MALAT1* overexpression and DGCR8 expression, suggesting that Drosha-DGCR8 complex (Microprocessor) controlled the abundance of Δsv-*MALAT1* in breast cancer as in HEK 293T cells (Macias *et al*, 2012). No such link was observed with Ago2, a second putative major regulator of *MALAT1* (Weinmann *et al*, 2009). We observed a positive link between Δsv-*MALAT1* overexpression and several interactors of *MALAT1*, in particular SFRS1 and SFRS3, confirming the involvement of *MALAT1* in the regulation of alternative splicing of pre-mRNA in nuclear speckle domains (Tripathi *et al*, 2010). We also identified a link between Δsv-*MALAT1* overexpression and several genes involved in DNA repair (*ATM, MSH2, XRCC1*). Only one study has recently suggested that *MALAT1* depletion could dysregulate ATM-CHK2 pathway in oesophageal squamous cell carcinoma (Hu *et al*, 2015). More interesting, we observed a link between Δsv-*MALAT1* expression and the major members (except *EZH2*) of Polycomb repressive complex PRC2, including *SUZ12, EED* and *JARID2*, but no (or little) link with the members of the Polycomb repressive complex PRC1 (i.e., *CBX4, CBX7* and *BMI1*), as well as the genes regulated by this complex: *PCNA* and *CCNE1* (Yang *et al*, 2011). In this regard, Yang *et al* (2011) showed a major role for *MALAT1* in the relocation of transcription units by the PRC2 complex in the three-dimensional space of the nucleus, to coordinate the gene-expression programs.

In conclusion, this study suggests that the lncRNA *MALAT1* (as the well-known lncRNA *HOTAIR*) is involved in breast cancer. These data revealed a complex expression pattern of various *MALAT1* transcript variants, and suggest that this pattern of expression should be taken into account when evaluating antitumoural drugs designed to target this lncRNA. Further studies are also necessary to elucidate roles of these different *MALAT1* transcript variants in breast tumourigenesis and their genetic (or epigenetic) dysregulation molecular mechanisms in this cancer.

## Figures and Tables

**Table 1:** Relationship between Δsv-*MALAT1*-spliced transcript levels and classical clinical biological parameters in a series of 446 breast cancer

		**Number of patients (%)**			
	**Total population (%)**	**Δsv-*****MALAT1*** **underexpression**	**Δsv-*****MALAT1*** **normal expression**	**Δsv-*****MALAT1*** **overexpression**	***P*****-value**^a^
Total	446 (100.0)	84 (18.8)	338 (75.8)	24 (5.4)	
**Age (years)**					
⩽50	94 (21.1)	25 (26.6)	67 (71.3)	2 (2.1)	**0.038**
>50	352 (78.9)	59 (16.8)	271 (77.0)	22 (6.3)	
**SBR histological grade**^b^^,^^c^					
I	57 (13.0)	4 (7.0)	49 (86.0)	4 (7.0)	**0.043**
II+III	380 (87.0)	79 (20.8)	282 (74.2)	19 (5.0)	
**Lymph node status**^d^					
0	117 (26.3)	17 (14.5)	90 (76.9)	10 (8.5)	**0.043**
1–3	231 (51.9)	39 (16.9)	182 (78.8)	10 (4.3)	
>3	97 (21.8)	27 (27.8)	66 (68.0)	4 (4.1)	
**Macroscopic tumour size**^e^					
⩽25 mm	218 (49.8)	28 (12.8)	174 (79.8)	16 (7.3)	**0.0023**
>25 mm	220 (50.2)	55 (25.0)	157 (71.4)	8 (3.6)	
**ERα status**					
Negative	115 (25.8)	37 (32.2)	76 (66.1)	2 (1.7)	**0.000062**
Positive	331 (74.2)	47 (14.2)	262 (79.2)	22 (6.6)	
**PR status**					
Negative	191 (42.8)	55 (28.8)	132 (69.1)	4 (2.1)	**0.0000051**
Positive	255 (57.2)	29 (11.4)	206 (80.8)	20 (7.8)	
**ERBB2 status**					
Negative	353 (79.1)	60 (17.0)	273 (77.3)	20 (5.7)	0.15 (NS)
Positive	93 (20.9)	24 (25.8)	65 (70.0)	4 (4.3)	
**Molecular subtypes**					
RH^−^ ERBB2^−^	68 (15.2)	22 (32.4)	46 (67.6)	0 (0)	**0.00074**
RH^−^ ERBB2^+^	42 (9.4)	14 (33.3)	26 (61.9)	2 (4.8)	
RH+ ERBB2^−^	285 (63.9)	38 (13.3)	227 (79.6)	20 (7.0)	
RH+ ERBB2^+^	51 (11.4)	10 (19.6)	39 (76.5)	2 (3.9)	
**PIK3CA mutation status**					
Wild type	299 (67.0)	69 (23.1)	215 (71.9)	15 (5.0)	**0.0049**
Mutated	147 (33.0)	15 (10.2)	123 (83.7)	9 (6.1)	
**MKI67 mRNA expression**					
Median	12.5 (0.80–117)	17.6 (0.86–117)	11.8 (0.80–94.5)	11.2 (0.93–47.1)	**0.00033**^f^
**EGFR mRNA expression**					
Median	0.22 (0.00–106)	0.13 (0.02–3.20)	0.23 (0.00–106)	0.20 (0.03–1.51)	**0.019**^f^

Abbreviations: ERα=oestrogen receptor-α ERBB2=human epidermal growth factor receptor 2; HR=hormone receptor; NS=not significant; PR=progesterone receptor.

The bold values are statistically significant (*P*-value<0.05).

a *χ*^2^-test.

b Scarff Bloom Richardson classification.

c Information available for 437 patients.

d Information available for 445 patients.

e Information available for 438 patients.

f Kruskal–Wallis's H test.

**Table 2:** Relationship between Δsv-*MALAT1*-mRNA and target-gene expression

**Genes**	**Normal breast tissues (*****n*****=10)**	**Breast tumours with low level of Δsv-*****MALA***(***n*****=20)**	**Breast tumours with high level of Δsv-*****MALAT1***(***n*****=20)**	***P*****-value**^a^	**ROC–AUC**
**Cell cycle control (*****n*****=7)**					
*MKI67*	1.0 (0.00–4.74)^b^	12.11 (1.88–25.62)^b^	13.01 (6.15–81.52)^b^	0.42 (NS)	0.575
*AURKA*	1.0 (0.17–2.73)	8.07 (2.79–27.11)	7.0 (4.28–123.17)	0.45 (NS)	0.570
*FOXM1*	1.0 (0.00–12.25)	10.91 (2.87–30.02)	13.74 (4.1–101.38)	0.40 (NS)	0.577
*PCNA*	1.0 (0.46–3.22)	2.72 (0.65–8.31)	3.59 (0.45–6.88)	0.24 (NS)	0.626
*CCNE1*	1.0 (0.00–6.82)	3.34 (0.90–26.50)	3.18 (1.14–52.07)	0.96 (NS)	0.505
*E2F1*	1.0 (0.44–3.08)	4.50 (1.00–8.60)	5.05 (1.76–51.10)	0.73 (NS)	0.532
*AURKB*	1.0 (0.00–16.86)	23.59 (2.90–37.43)	15.38 (6.77–160.48)	0.75 (NS)	0.47
**Cell migration (*****n*****=5)**					
*RHOB*	1.0 (0.48–5.75)	0.88 (0.19–1.36)	1.13 (0.36–65.33)	**0.015**	0.724
*PLAU*	1.0 (0.42–2.29)	1.76 (0.31–12.59)	2.86 (0.84–33.28)	**0.017**	0.72
*MMP14*	1.0 (0.69–1.52)	1.31 (0.05–6.21)	2.52 (0.30–39.17)	**0.0087**	0.743
*RHOA*	1.0 (0.42–3.34)	1.36 (0.22–3.85)	1.59 (0.22–6.39)	0.38 (NS)	0.581
*MMP13*	1.0 (0.00–4.69)	45.38 (1.33–688.61)	38.70 (2.62–250.99)	0.83 (NS)	0.48
**Polycomb repressive complex 1 (*****n*****=3)**					
*CBX7*	1.0 (0.39–1.62)	0.36 (0.05–0.95)	0.43 (0.15–2.43)	**0.045**	0.685
*CBX4*	1.0 (0.41–3.80)	1.46 (0.79–2.98)	1.55 (0.00–3.10)	0.91 (NS)	0.501
*BMI1*	1.0 (0.59–1.47)	1.42 (0.17–4.91)	1.98 (0.66–6.43)	**0.014**	0.728
**Polycomb repressive complex 2 (*****n*****=5)**					
*SUZ12*	1.0 (0.56–1.25)	1.19 (0.53–1.93)	1.63 (0.92–2.99)	**0.00084**	0.793
*JARID2*	1.0 (0.83–1.46)	1.19 (0.71–1.83)	1.89 (0.51–4.13)	**0.0015**	0.794
*EED*	1.0 (0.58–1.84)	1.02 (0.18–2.61)	1.36 (0.19–3.13)	**0.0036**	0.769
*TUG1*	1.0 (0.79–3.06)	0.73 (0.35–2.01)	1.16 (0.34–1.98)	**0.044**	0.686
*EZH2*	1.0 (0.48–2.32)	4.58 (1.36–12.70)	5.99 (2.76–14.64)	0.15 (NS)	0.632
**EMT (*****n*****=7)**					
*CDH1*	1.0 (0.33–1.79)	0.91 (0.06–2.74)	1.28 (0.23–5.55)	0.051 (NS)	0.68
*VIM*	1.0 (0.42–3.11)	0.22 (0.09–0.70)	0.31 (0.10–2.16)	0.22 (NS)	0.614
*ZEB2*	1.0 (0.44–5.58)	0.30 (0.12–0.64)	0.32 (0.14–2.35)	0.60 (NS)	0.549
*ZEB1*	1.0 (0.43–4.08)	0.31 (0.19–1.47)	0.44 (0.16–1.09)	0.34 (NS)	0.587
*SNAI2*	1.0 (0.43–1.50)	0.23 (0.05–0.82)	0.24 (0.11–1.41)	0.52 (NS)	0.56
*TWIST1*	1.0 (0.53–3.62)	0.30 (0.08–1.16)	0.32 (0.07–3.83)	0.63 (NS)	0.545
*SNAI1*	1.0 (0.34–7.88)	0.83 (0.06–2.66)	0.89 (0.20–6.03)	0.80 (NS)	0.524
**Apoptosis (*****n*****=6)**					
*BIRC6*	1.0 (0.74–3.10)	0.85 (0.47–1.23)	1.13 (0.52–1.75)	**0.00017**	0.841
*BAX*	1.0 (0.41–8.88)	1.38 (0.62–3.68)	1.88 (0.80–8.26)	**0.02**	0.715
*BIRC2*	1.0 (0.73–3.61)	0.62 (0.30–1.18)	0.82 (0.26–1.99)	**0.044**	0.686
*BIRC4*^c^	0.0 (0.00–1.16)	1.09 (0.00–6.88)	2.16 (0.00–8.59)	0.12 (NS)	0.645
*BCL2L1*	1.0 (0.48–3.76)	1.19 (0.65–2.19)	1.47 (0.52–4.69)	0.22 (NS)	0.612
*BCL2*	1.0 (0.34–6.13)	0.85 (0.08–3.75)	0.78 (0.24–2.43)	0.82 (NS)	0.521
**DNA repair (*****n*****=6)**					
*ATM*	1.0 (0.67–1.66)	0.60 (0.40–1.87)	1.36 (0.55–2.24)	**0.00007**	0.868
*MSH2*	1.0 (0.66–1.55)	0.98 (0.60–1.57)	1.40 (0.83–2.95)	**0.0003**	0.834
*BRCA1*	1.0 (0.00–2.56)	2.23 (0.05–13.56)	3.33 (0.00–8.85)	0.11 (NS)	0.649
*BRCA2*	1.0 (0.27–2.49)	3.12 (0.86–7.82)	3.87 (0.00–9.57)	0.19 (NS)	0.631
*RAD51*	1.0 (0.0–2.00)	7.55 (1.62–18.9)	5.29 (1.35–21.6)	0.37 (NS)	0.417
*XRCC1*	1.0 (0.70–1.30)	0.78 (0.53–1.34)	1.41 (0.78–2.84)	**0.00001**	0.908
**Regulators of MALAT1 (*****n*****=6)**					
*DGCR8*	1.0 (0.70–2.59)	0.80 (0.40–1.71)	1.12 (0.80–5.05)	**0.000083**	0.864
*AGO2*	1.0 (0.57–4.50)	0.8 (0.36–2.64)	0.89 (0.33–5.87)	0.26 (NS)	0.604
*SFRS1*	1.0 (0.66–4.32)	1.13 (0.87–1.68)	1.51(0.98–2.80)	**0.00097**	0.805
*SFRS3*	1.0 (0.65–3.93)	1.06 (0.69–1.87)	1.30 (0.78–3.05)	**0.036**	0.694
*SFRS2*	1.0 (0.60–2.76)	1.52 (0.88–2.24)	1.89 (0.81–4.04)	0.18 (NS)	0.624
*UHMK1*	1.0 (0.59–5.09)	1.48 (0.93–2.74)	2.04 (0.95–3.88)	**0.0094**	0.74
**MALAT1-inducible genes (*****n*****=3)**					
*ROBO1*	1.0 (0.54–1.54)	0.36 (0.13–4.14)	0.52 (0.14–1.33)	0.14 (NS)	0.635
*MCAM*	1.0 (0.57–33.8)	0.52 (0.22–0.81)	0.61 (0.22–10.9)	0.21 (NS)	0.616
*IFI44*	1.0 (0.34–9.38)	1.92 (0.06–29.23)	2.63 (0.11–30.15)	0.43 (NS)	0.572

Abbreviations: AUC=area under the curve; NS=not significant; ROC=receiver operating curves. Values in bold correspond to significant *P*-values (*P*<0.05).

a Kruskal–Wallis *H*-test.

b Median (range) of gene mRNA levels ; the mRNA values of the samples were normalised such that the median of the 10 normal breast tissues mRNA values was 1.

c The mRNA values of the samples were normalised such that a Ct value of 35 was 1.

**Table 3:** Relationship between levels of Δsv*-MALAT1* and FL*-MALAT1* and a panel of RTK/MAPK/PI3K proteins in a panel of 143 breast tumours

	**Δsv*****-MALAT1***		**FL*****-MALAT1***	
	***r***^a^	***P*****-value**^a^	***r***^a^	***P*****-value**^a^
**RTK proteins (*****n*****=9)**				
EGFR	0.068	NS	0.033	NS
p.EGFR.Thr669	0.008	NS	−0.047	NS
p.EGFR.Tyr1173	0.036	NS	0.052	NS
Her2/ErbB2	0.091	NS	−0.032	NS
Her3/ErbB3	−0.128	NS	0.000	NS
p.Her3/Erbb3.Tyr1289	−0.060	NS	−0.020	NS
p.Her4.Tyr1162	0.108	NS	0.018	NS
Met	−0.025	NS	−0.029	NS
p.Met.Tyr1234.1235	−0.064	NS	−0.026	NS
**MAPK pathway proteins (*****n*****=4)**				
MEK1/2	−0.029	NS	−0.073	NS
p.MEK1/2.Ser217/221	0.113	NS	−0.047	NS
p44.42.MAPK	0.116	NS	0.150	NS
p.p44.42.MAPK.Thr202.Tyr204	0.127	NS	0.030	NS
**PI3K/AKT pathway proteins (*****n*****=15)**				
PTEN	0.101	NS	0.050	NS
p.PTEN.Ser380.Thr382.383	−0.018	NS	−0.026	NS
INPP4b	0.001	NS	0.042	NS
Akt1	0.030	NS	−0.017	NS
p.Akt1.Ser473	0.119	NS	−0.032	NS
Akt2	−0.102	NS	0.028	NS
mTor	−0.155	NS	−0.066	NS
p.mTor.Ser2448	−0.077	NS	0.008	NS
FOXO1	−**0.194**	**0.0210**	−**0.196**	**0.0180**
TSC2	−0.075	NS	0.003	NS
p70.S6.Kinase	−**0.172**	**0.0400**	0.099	NS
p.p70.S6.Kinase.Thr389	−**0.225**	**0.0070**	0.073	NS
S6.Ribosomal.protein	−0.034	NS	0.075	NS
p.S6.Ribosomal.protein.Ser235.236	−0.095	NS	0.006	NS
p.S6.Ribosomal.protein.Ser240.244	−**0.201**	**0.0160**	−0.024	NS

Abbreviations: Akt=protein kinase B, also known as Akt; EGFR=epidermal growth factor receptor; MAPK=mitogen-activated protein kinase; mTor=mechanistic target of rapamycin; NS=not significant; PI3K=phosphoinositide 3-kinase; PTEN=phosphatase and tensin homologue.

a Spearman's rank correlation test.
