# Supplementary material for: Prognostic value of a newly identified MALAT1 alternatively spliced transcript in breast cancer
Source: Br J Cancer. 2016 May 12;114(12):1395–404. doi: 10.1038/bjc.2016.123 (PMC4984455; doi:10.1038/bjc.2016.123)
Supplement: Supplementary Figure 1 [file bjc2016123x1.ppt]

## Slide 1
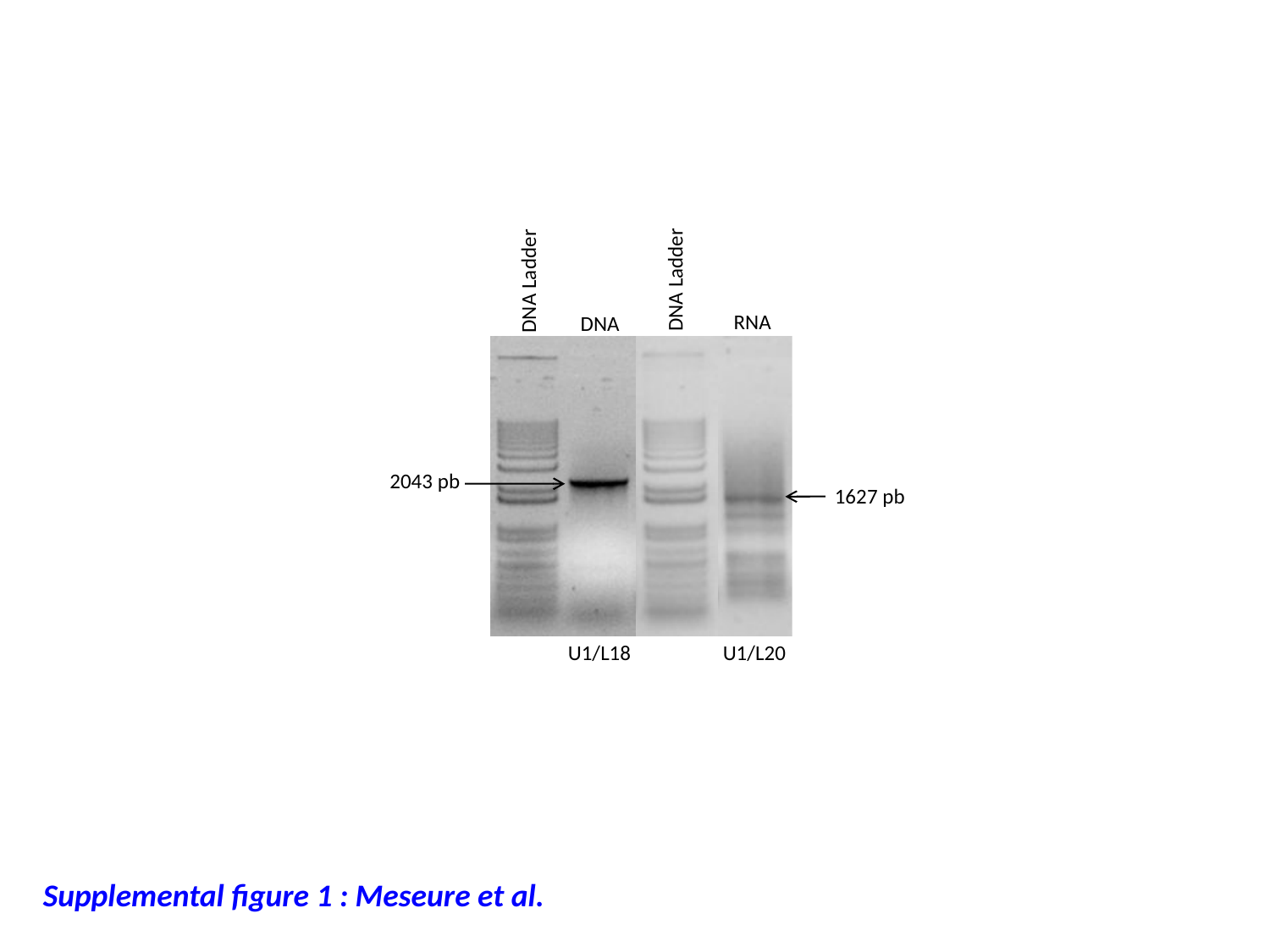

DNA Ladder
DNA Ladder
RNA
DNA
2043 pb
1627 pb
U1/L18
U1/L20
Supplemental figure 1 : Meseure et al.
Supplemental figure 2 : Meseure et al.
Supplemental figure 2 : Meseure et al.
Supplemental figure 2 : Meseure et al.
