# Supplementary figures and images for: Prognostic value of a newly identified MALAT1 alternatively spliced transcript in breast cancer
Source: Br J Cancer. 2016 May 12;114(12):1395–404. doi: 10.1038/bjc.2016.123 (PMC4984455; doi:10.1038/bjc.2016.123)

## Slide 1
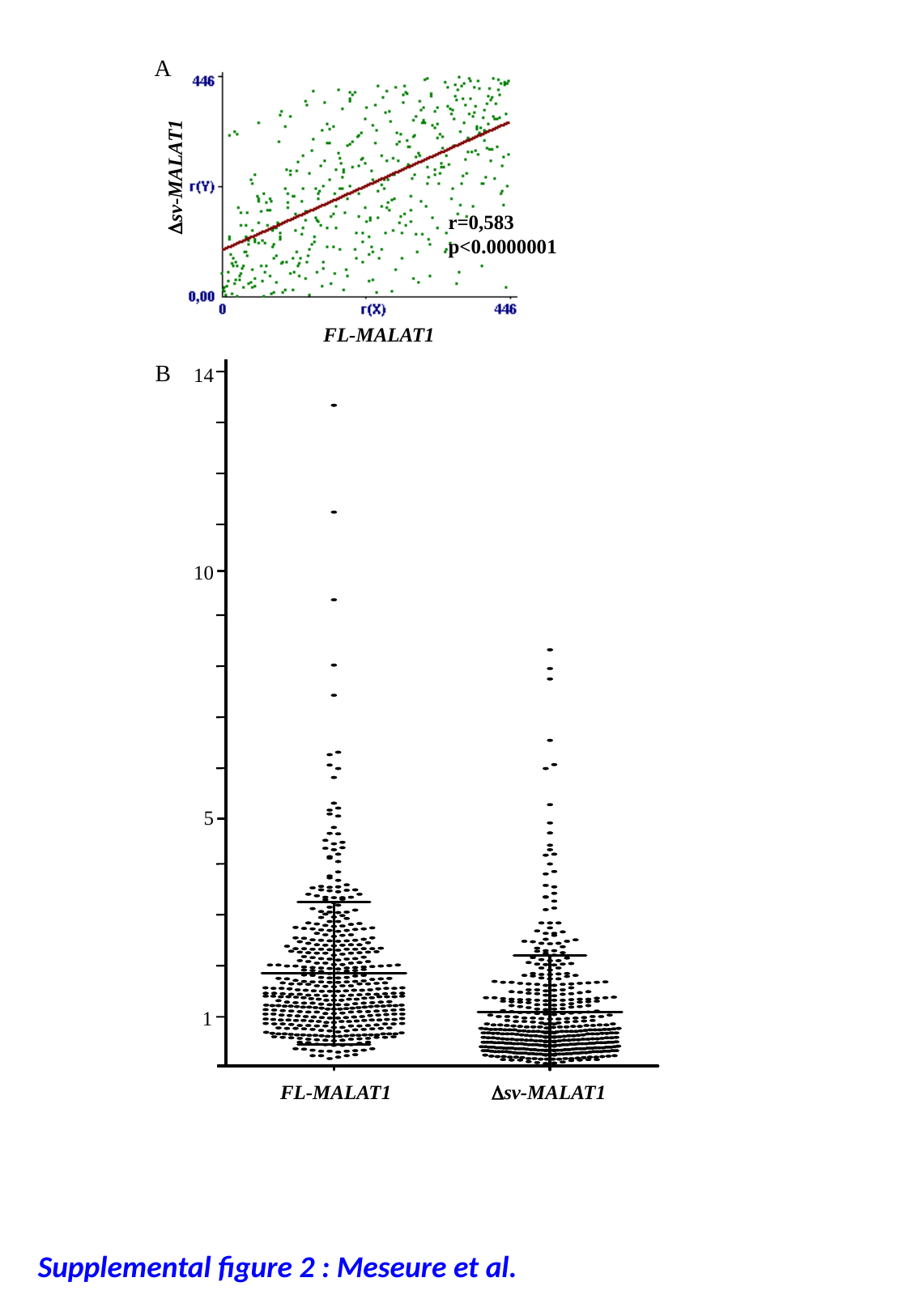

A
Δsv-MALAT1
r=0,583
p<0.0000001
FL-MALAT1
B
14
10
5
1
FL-MALAT1
Δsv-MALAT1
Supplemental figure 2 : Meseure et al.

Supplement: Supplementary Figure 2 [file bjc2016123x2.ppt]
