# Supplementary material for: Prognostic value of a newly identified MALAT1 alternatively spliced transcript in breast cancer
Source: Br J Cancer. 2016 May 12;114(12):1395–404. doi: 10.1038/bjc.2016.123 (PMC4984455; doi:10.1038/bjc.2016.123)
Supplement: Supplementary Table 1 [file bjc2016123x3.doc]

**Supplemental Table 1: Pathological and clinical characteristics of patients in relation to metastasis free survival (MFS)**

|  | Number of patients (%) | Number with metastases (%) | MFS  *p*-value *^a^* |
| --- | --- | --- | --- |
|  |  |  |  |
| *Total* | 446 (100.0) | 176 (39.5) |  |
|  |  |  |  |
| *Age*  ≤50  >50 | 94 (21.1)  352 (78.9) | 37 (39.4)  139 (39.5) | 0.96 (NS) |
| *SBR histological grade* b, c  I  II + III | 57 (13.0)  380 (87.0) | 11 (19.3)  161 (42.4) | **0.00073** |
| *Lymph node status* d  0  1-3  >3 | 117 (26.3)  231 (51.9)  97 (21.8) | 36 (30.8)  80 (34.6)  60 (61.9) | **0.00000036** |
| *Macroscopic tumor size* e  ≤25mm  >25mm | 218 (49.8)  220 (50.2) | 66 (30.3)  109 (49.5) | **0.0000095** |
| *ERα status*  Negative  Positive | 115 (25.8)  331 (74.2) | 47 (40.9)  129 (39.0) | 0.18 (NS) |
| *PR status*  Negative  Positive | 191 (42.8)  255 (57.2) | 83 (43.5)  93 (36.5) | **0.028** |
| *ERBB2 status*  Negative  Positive | 353 (79.1)  93 (20.9) | 137 (38.8)  39 (41.9) | 0.45 (NS) |
| *Molecular subtypes*  HR- ERBB2-  HR- ERBB2+  HR+ ERBB2-  HR+ ERBB2+ | 68 (15.2)  42 (9.4)  285 (63.9)  51 (11.4) | 26 (38.2)  20 (47.6)  111 (38.9)  19 (37.3) | 0.20 (NS) |
| *PIK3CA mutation status*  wild type  mutated | 299 (67.0)  147 (33.0) | 124 (41.5)  52 (35.4) | 0.12 (NS) |

Abbreviations: ERα: oestrogen receptor alpha; PR: progesterone receptor; ERBB2: human epidermal growth factor receptor 2; HR: hormone receptor.

a Log-rank test : the bold values are statistically significant (*p*-value<0.05). NS: not significant. b Scarff Bloom Richardson classification. c Information available for 437 patients. d Information available for 445 patients. e Information available for 438 patients.
