# Supplementary material for: Prognostic value of a newly identified MALAT1 alternatively spliced transcript in breast cancer
Source: Br J Cancer. 2016 May 12;114(12):1395–404. doi: 10.1038/bjc.2016.123 (PMC4984455; doi:10.1038/bjc.2016.123)
Supplement: Supplementary Table 3 [file bjc2016123x5.doc]

**Supplemental Table 3: Relationship between *MALAT1* transcript levels and classical clinical biological parameters in a series of 446 breast cancer**

|  |  | Number of patients (%) | | |  |
| --- | --- | --- | --- | --- | --- |
|  | Total population (%) | *MALAT1* under expression | *MALAT1* normal expression | *MALAT1* over expression | *p*-value ^a^ |
|  |  |  |  |  |  |
| *Total* | 446 (100.0) | 13 (2.9) | 370 (83.0) | 63 (14.1) |  |
|  |  |  |  |  |  |
| *Age*  ≤50  >50 | 94 (21.1)  352 (78.9) | 4 (4.3)  9 (2.6) | 79 (84.0)  291 (82.7) | 11 (11.7)  52 (14.8) | 0.54 (NS) |
| *SBR histological grade* b, c  I  II + III | 57 (13.0)  380 (87.0) | 2 (3.5)  11 (2.9) | 49 (86.0)  315 (82.9) | 6 (10.5)  54 (14.2) | 0.74 (NS) |
| *Lymph node status* d  0  1-3  >3 | 117 (26.3)  231 (51.9)  97 (21.8) | 4 (3.4)  5 (2.2)  3 (3.1) | 92 (78.6)  198 (85.7)  80 (82.5) | 21 (17.9)  28 (12.1)  14 (14.4) | 0.58 (NS) |
| *Macroscopic tumor size* e  ≤25mm  >25mm | 218 (49.8)  220 (50.2) | 4 (1.8)  8 (3.6) | 179 (82.1)  186 (84.5) | 35 (16.1)  26 (11.8) | 0.25 (NS) |
| *ERα status*  Negative  Positive | 115 (25.8)  331 (74.2) | 9 (7.8)  4 (1.2) | 101 (87.8)  269 (81.3) | 5 (4.3)  58 (17.5) | **0.000015** |
| *PR status*  Negative  Positive | 191 (42.8)  255 (57.2) | 11 (5.8)  2 (0.8) | 162 (84.8)  208 (81.6) | 18 (9.4)  45 (17.6) | **0.00079** |
| *ERBB2 status*  Negative  Positive | 353 (79.1)  93 (20.9) | 13 (3.7)  0 (0) | 290 (82.2)  80 (86.0) | 50 (14.2)  13 (14.0) | 0.17 (NS) |
| *Molecular subtypes*  HR- ERBB2-  HR- ERBB2+  HR+ ERBB2-  HR+ ERBB2+ | 68 (15.2)  42 (9.4)  285 (63.9)  51 (11.4) | 9 (13.2)  0 (0)  4 (1.4)  0 (0) | 57 (83.8)  39 (92.9)  233 (81.8)  41 (80.4) | 2 (2.9)  3 (7.1)  48 (16.8)  10 (19.6) | **0.00000075** |
| *PIK3CA mutation status*  wild type  mutated | 299 (67.0)  147 (33.0) | 9 (3.0)  4 (2.7) | 250 (83.6)  120 (81.6) | 40 (13.4)  23 (15.6) | 0.81 (NS) |
| *MKI67 mRNA expression*  median | 12.5 (0.80-117) | 13.7 (6.90-40.6) | 12.4 (0.80-117) | 12.2 (0.98-81.5) | 0.66 (NS) ^f^ |
| *EGFR mRNA expression*  median | 0.22 (0.00-106) | 0.52 (0.07-3.20) | 0.22 (0.00-106) | 0.20 (0.03-7.56) | 0.27 (NS) ^f^ |

Abbreviations: ERα: oestrogen receptor alpha; PR: progesterone receptor; ERBB2: human epidermal growth factor receptor 2; HR: hormone receptor.

The bold values are statistically significant (*p*-value<0.05). NS: not significant. a Chi-squared test. b Scarff Bloom Richardson classification. c Information available for 437 patients. d Information available for 445 patients. e Information available for 438 patients. f Kruskal Wallis’s H Test.
