# Supplementary material for: Prognostic value of a newly identified MALAT1 alternatively spliced transcript in breast cancer
Source: Br J Cancer. 2016 May 12;114(12):1395–404. doi: 10.1038/bjc.2016.123 (PMC4984455; doi:10.1038/bjc.2016.123)
Supplement: Supplementary Table 5 [file bjc2016123x7.doc]

**Supplemental Table 5: Relationship between ∆*sv-MALAT1* spliced transcripts levels (using U2/L2 primers) and classical clinical biological parameters in a series of 446 breast cancer.**

|  |  | Number of patients (%) | | |  |
| --- | --- | --- | --- | --- | --- |
|  | Total population (%) | ∆*sv*−*MALAT1* under- expression | ∆*sv*−*MALAT1* normal  expression | ∆*sv*−*MALAT1* over  expression | *p*-value ^a^ |
|  |  |  |  |  |  |
| *Total* | 446 (100.0) | 99 (22.2) | 306 (68.6) | 41 (9.2) |  |
|  |  |  |  |  |  |
| *Age*  ≤50  >50 | 94 (21.1)  352 (78.9) | 30 (31.9)  69 (19.6) | 63 (67.0)  243 (69.0) | 1 (1.1)  40 (11.4) | **0.0012** |
| *SBR histological grade* b, c  I  II + III | 57 (13.0)  380 (87.0) | 5 (8.8)  93 (24.5) | 46 (80.7)  254 (66.8) | 6 (10.5)  33 (8.7) | **0.029** |
| *Lymph node status* d  0  1-3  >3 | 117 (26.3)  231 (51.9)  97 (21.8) | 21 (17.9)  49 (21.2)  28 (28.9) | 81 (69.2)  164 (71.0)  61 (62.9) | 15 (12.8)  18 (7.8)  8 (8.2) | 0.20 (NS) |
| *Macroscopic tumor size* e  ≤25mm  >25mm | 218 (49.8)  220 (50.2) | 38 (17.4)  60 (27.3) | 159 (72.9)  143 (65.0) | 21 (9.6)  17 (7.7) | **0.044** |
| *ERα status*  Negative  Positive | 115 (25.8)  331 (74.2) | 40 (34.8)  59 (17.8) | 68 (59.1)  238 (71.9) | 7 (6.1)  34 (10.3) | **0.00077** |
| *PR status*  Negative  Positive | 191 (42.8)  255 (57.2) | 59 (30.9)  40 (15.7) | 121 (63.4)  185 (72.5) | 11 (5.8)  30 (11.8) | **0.00027** |
| *ERBB2 status*  Negative  Positive | 353 (79.1)  93 (20.9) | 75 (21.2)  24 (25.8) | 244 (69.1)  62 (66.7) | 34 (9.6)  7 (7.5) | 0.58 (NS) |
| *Molecular subtypes*  RH- ERBB2-  RH- ERBB2+  RH+ ERBB2-  RH+ ERBB2+ | 68 (15.2)  42 (9.4)  285 (63.9)  51 (11.4) | 25 (36.8)  14 (33.3)  50 (17.5)  10 (19.6) | 40 (58.8)  24 (57.1)  204 (71.6)  38 (74.5) | 3 (4.4)  4 (9.5)  31 (10.9)  3 (5.9) | **0.0082** |
| *PIK3CA mutation status*  wild type  mutated | 299 (67.0)  147 (33.0) | 76 (25.4)  23 (15.6) | 198 (66.2)  108 (73.5) | 25 (8.4)  16 (10.9) | 0.057 (NS) |
| *MKI67 mRNA expression*  median | 12.5 (0.80-117) | 16.6 (2.09-75.1) | 11.6 (0.80-117) | 11.3 (0.85-62.6) | **0.00018** ^f^ |
| *EGFR mRNA expression*  median | 0.22 (0.00-106) | 0.15 (0.02-3.20) | 0.24 (0.00-106) | 0.22 (0.04-2.56) | **0.0044** ^f^ |

Abbreviations: ERα: oestrogen receptor alpha; PR: progesterone receptor; ERBB2: human epidermal growth factor receptor 2; HR: hormone receptor.

The bold values are statistically significant (*p*-value<0.05). NS: not significant. a Chi-squared test. b Scarff Bloom Richardson classification. c Information available for 437 patients. d Information available for 445 patients. e Information available for 438 patients. f Kruskal Wallis’s H Test.
