# Supplementary material for: Prognostic value of a newly identified MALAT1 alternatively spliced transcript in breast cancer
Source: Br J Cancer. 2016 May 12;114(12):1395–404. doi: 10.1038/bjc.2016.123 (PMC4984455; doi:10.1038/bjc.2016.123)
Supplement: Supplementary Figure Legends [file bjc2016123x9.doc]

**Supplemental figure 1**: Visualization of *FL-MALAT1* (on genomic DNA) and ∆sv-*MALAT1* (on total RNA) RT-PCR products by agarose gel electrophoresis. Using primer pair U1/L20 with one of the two primers (U20) placed at the junction of the 119-pb spliced region, we observed only a unique RT-PCR product showing both 119-bp and 243-bp deletions (1627-pb), but not the RT-PCR product only showing the 119-bp deletion (expected size of 1870-pb =1627-pb + 243-pb).

**Supplemental Figure 2:** (A) Spearman rank correlation test between *FL-MALAT1* and ∆*sv-MALAT1*. (B) Scatter dot plot of qRT-PCR data for *FL-MALAT1* and ∆*sv-MALAT1* in the series of 446 breast cancer.
